# Mechanical inhibition of isolated V_o_ from V/A-ATPase for proton conductance

**DOI:** 10.7554/eLife.56862

**Published:** 2020-07-08

**Authors:** Jun-ichi Kishikawa, Atsuko Nakanishi, Aya Furuta, Takayuki Kato, Keiichi Namba, Masatada Tamakoshi, Kaoru Mitsuoka, Ken Yokoyama

**Affiliations:** 1 Department of Molecular Biosciences, Kyoto Sangyo University, Kamigamo-MotoyamaKyotoJapan; 2 Institute for Protein Research, Osaka UniversitySuitaJapan; 3 Research Center for Ultra-High Voltage Electron Microscopy, Osaka University, Research Center for Ultra-High Voltage Electron Microscopy, MihogaokaOsakaJapan; 4 Graduate School of Frontier Biosciences, Osaka UniversitySuitaJapan; 5 RIKEN Center for Biosystems Dynamics Research and SPring-8 CenterSuitaJapan; 6 JEOL YOKOGUSHI Research Alliance Laboratories, Osaka UniversitySuitaJapan; 7 Department of Molecular Biology, Tokyo University of Pharmacy and Life Sciences, Horinouchi, HachiojiTokyoJapan; Weill Cornell MedicineUnited States; Michigan State UniversityUnited States

**Keywords:** single particle cryo-em, rotary ATPase, V/A-ATPase, ATP synthase, Other

## Abstract

V-ATPase is an energy converting enzyme, coupling ATP hydrolysis/synthesis in the hydrophilic V_1_ domain, with proton flow through the V_o_ membrane domain, via rotation of the central rotor complex relative to the surrounding stator apparatus. Upon dissociation from the V_1_ domain, the V_o_ domain of the eukaryotic V-ATPase can adopt a physiologically relevant auto-inhibited form in which proton conductance through the V_o_ domain is prevented, however the molecular mechanism of this inhibition is not fully understood. Using cryo-electron microscopy, we determined the structure of both the *holo* V/A-ATPase and isolated V_o_ at near-atomic resolution, respectively. These structures clarify how the isolated V_o_ domain adopts the auto-inhibited form and how the *holo* complex prevents formation of the inhibited V_o_ form.

## Introduction

**Figure 1.**
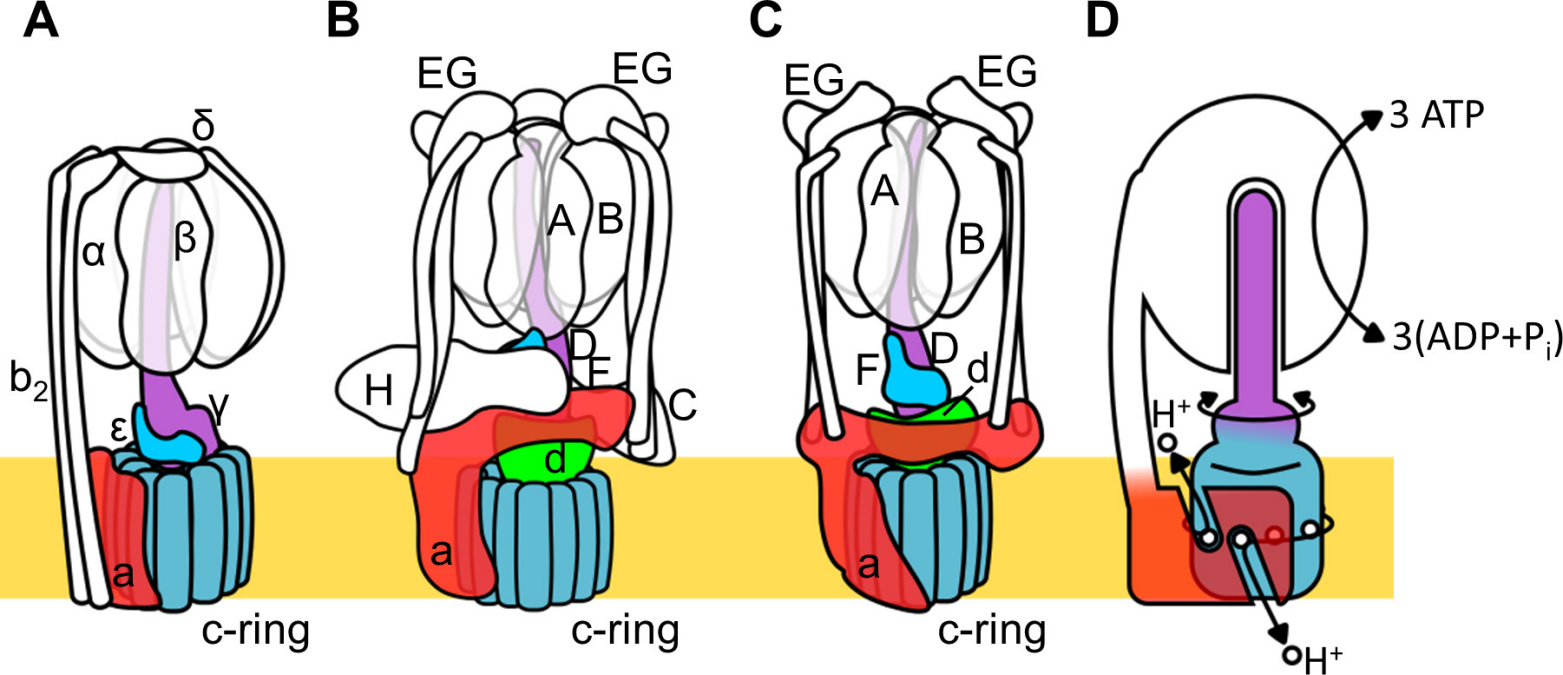
Schematic of rotary ATPase/synthases and the rotary catalytic mechanism. (**A**) Bacterial F_o_F_1_, (**B**) yeast V-ATPase, (**C**) *Tth* V/A-ATPase, (**D**) a schematic model of the rotary catalytic mechanism. The subunits of the central rotor complex are colored as follows: c-ring, dark blue; a-subunit, red; central axis, purple and cyan; and d-subunit, green.

Rotary ATPase/ATP synthases, roughly classified into F type and V type ATPases, are marvelous, tiny rotary machines (Yokoyama and Imamura, 2005; Kinosita, 2012; Forgac, 2007; Yoshida et al., 2001; Kühlbrandt, 2019). These rotary motor proteins share a basic molecular architecture composed of a central rotor complex and surrounding stator apparatus. These proteins function to couple ATP hydrolysis/synthesis in the hydrophilic F_1_/V_1_ moiety with proton translocation through the membrane embedded hydrophobic F_o_/V_o_ moiety by rotation of the central rotor complex relative to the surrounding stator apparatus, via a rotary catalytic mechanism (Figure 1; Kinosita, 2012; Forgac, 2007; Yoshida et al., 2001; Kühlbrandt, 2019; Guo and Rubinstein, 2018).

**Figure 1—figure supplement 1.**
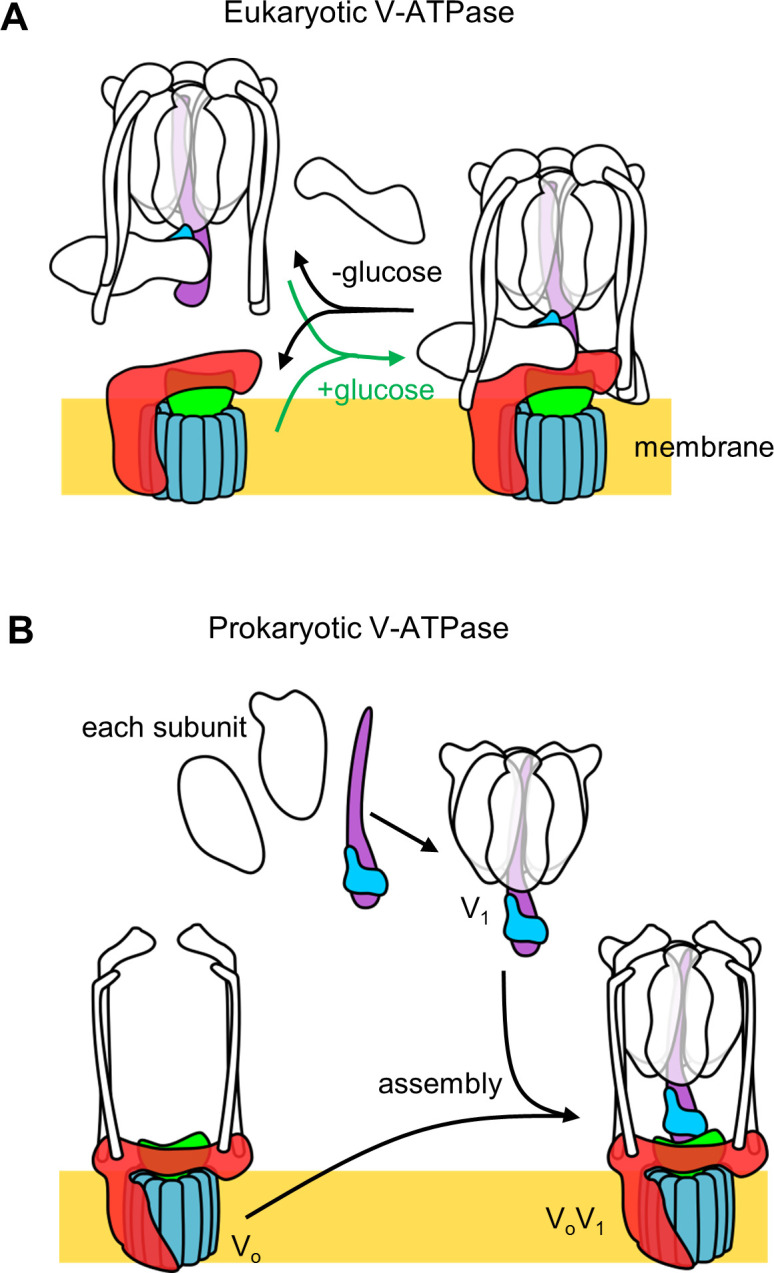
Schematic representation of reversible dissociation of the V_1_ domain induced by glucose depletion in yeast (**A**), and the assembly pathway of the *holo* V/A-ATPase in *Tth* cells (**B**).

Thus, both F and V type ATPases are basically capable of either ATP synthesis coupled to the proton motive force (*pmf*) driven by the membrane potential or proton pumping powered by ATP hydrolysis. The F type ATPase (F-ATPase, or F_o_F_1_) in mitochondria functions as an ATP synthase coupled to respiration, whilst in some bacteria the complex can function as an ATP dependent proton pump (Shibata et al., 1992; Kullen and Klaenhammer, 1999).

The V type ATPase (V-ATPase, or V_o_V_1_) resides mainly in the membranes of acidic vesicles in eukaryote cells, functioning as a proton pump using a rotary catalytic mechanism (Forgac, 2007; Yokoyama et al., 2003; Imamura et al., 2003). Eukaryotic V-ATPases probably evolved from the prokaryotic enzymes (Gogarten et al., 1989; Tsutsumi et al., 1991), termed Archaeal ATPases or V/A-ATPases (Forgac, 2007; Kühlbrandt and Davies, 2016). The V/A-ATPase from a thermophilic bacterium, *Thermus thermophilus* (*Tth* V/A-ATPase) is a rotary ATPase that has been well characterized using both structure and single molecular observation studies (Yokoyama and Imamura, 2005; Yokoyama et al., 2003; Imamura et al., 2003; Iwata et al., 2004; Makyio et al., 2005; Toei et al., 2007; Nakanishi et al., 2018; Schep et al., 2016). The overall structure of *Tth* V/A-ATPase closely resembles that of the eukaryotic V-ATPase although it lacks some of the accessary subunits of the eukaryotic enzyme (Figure 1B,C). The *Tth* V_1_ moiety is composed of four subunits with a stoichiometry of A_3_B_3_D_1_F_1_ and it is responsible for ATP synthesis or hydrolysis (Yokoyama et al., 1998; Yokoyama et al., 1990). Upon dissociation from V_o_, the isolated V_1_ moiety displays only ATP hydrolysis activity accompanied by rotation of the DF shaft. The *Tth* V_o_ moiety, responsible for proton translocation across the membrane, contains a central rotor complex (*d*_1_*c*_12_) and stator apparatus made up of the *a* subunit and two EG peripheral stalks (*a*_1_E_2_G_2_). In the *holo Tth* V/A-ATPase, *pmf* drives rotation of the *d*_1_*c*_12_ rotor complex relative to the surrounding stator, resulting in rotation of the entire central rotor complex (D_1_F_1_*d*_1_*c*_12_) and inducing sequential conformation changes in the A_3_B_3_ catalytic hexamer to produce three ATP molecules from ADP and inorganic phosphates per one rotation (Figure 1D).

The eukaryotic V-ATPase is regulated by a unique mechanism involving dissociation/association of V_1_, that is likely to be a key factor in controlling the pH of acidic vesicles (Kane and Parra, 2000; Sharma et al., 2019; Toei et al., 2010). In yeast, glucose depletion in the culture medium induces dissociation of the V_1_ domain from V_o_ domain, resulting in reduced proton pumping activity of the V-ATPase (Figure 1—figure supplement 1A). It is likely that dissociated V_o_ loses the ability to translocate protons as a result of auto-inhibition (Couoh-Cardel et al., 2015; Qi and Forgac, 2008). In the structure of dissociated yeast V_o_, the hydrophilic region of the *a* subunit (*a*_sol_) changes its conformation to prevent rotation of the rotor complex (Roh et al., 2018; Mazhab-Jafari et al., 2016). The yeast *a*_sol_ region lies in close proximity to the *d* subunit, the rotor region of the isolated yeast V_o_ component. Both the *a*_sol_ region and *d* subunit represent the trademarks of the V-ATPase family lacking in F-ATPases (see Figure 1A–C; Iwata et al., 2004). Thus, the *a*_sol_ region and the *d* subunit appear to be crucial in stabilizing the auto-inhibition structure of the dissociated V_o_ domain.

A regulatory dissociation/association mechanism has not been reported for the bacterial V/A-ATPase, however, reconstitution experiments suggest an assembly pathway for the *holo* complex, in which cytosolic V_1_ associates with membrane V_o_ (Figure 1—figure supplement 1B; Kishikawa and Yokoyama, 2012). Thus, proton leak through the V_o_ domain in *Tth* membranes may also be blocked by an autoinhibition mechanism similar to that in the eukaryotic enzyme. Indeed, the *Tth* V/A-ATPase and eukaryotic V-ATPase share very similar structures with V_o_ moieties comprising the *a* and *d* subunits in addition to the c ring.

Structural analysis of several subunits and subcomplexes of V/A-ATPases has been successfully carried out (Iwata et al., 2004; Makyio et al., 2005; Toei et al., 2007; Nagamatsu et al., 2013; Murata et al., 2005). Recent advances of single particle cryogenic microscopy (cryoEM) have facilitated structural analysis of the entire *holo* complexes of prokaryotic and eukaryotic V-ATPases in several rotational states (Nakanishi et al., 2018; Zhou and Sazanov, 2019; Vasanthakumar et al., 2019). While several structures of the isolated yeast V_o_ have been reported (Roh et al., 2018; Mazhab-Jafari et al., 2016; Vasanthakumar et al., 2019), a high resolution structure of the isolated *Tth* V_o_ is currently unavailable, limiting understanding of the mechanism of enzyme inhibition.

Here, we report a cryoEM structure of isolated *Tth* V_o_ at 3.9 Å resolution. The V_o_ structure reveals that the *a*_sol_ region and *d* subunit adopt distinct conformation, appropriate for inhibiting rotation of *d*_1_*c*_12_ relative to the stator *a* subunit. This conformation is different from that seen in the V_o_ moiety of the complete *Tth* V/A-ATPase. Biochemical analysis using *Tth* V_o_ reconstituted into liposomes supports inhibition of proton conductance of isolated V_o_ with a threshold membrane potential. Our results indicate that bacterial and eukaryotic V_o_ domains use a similar mechanism for auto-inhibition of proton conductance. This mechanism prevents proton leak from *Tth* cells through an intermediate assembly of the V_o_ domain of *holo* V/A-ATPase under physiological conditions.

## Results

### CryoEM structures of the isolated V_o_ domain and *holo Tth* V/A-ATPase

**Figure 2.**
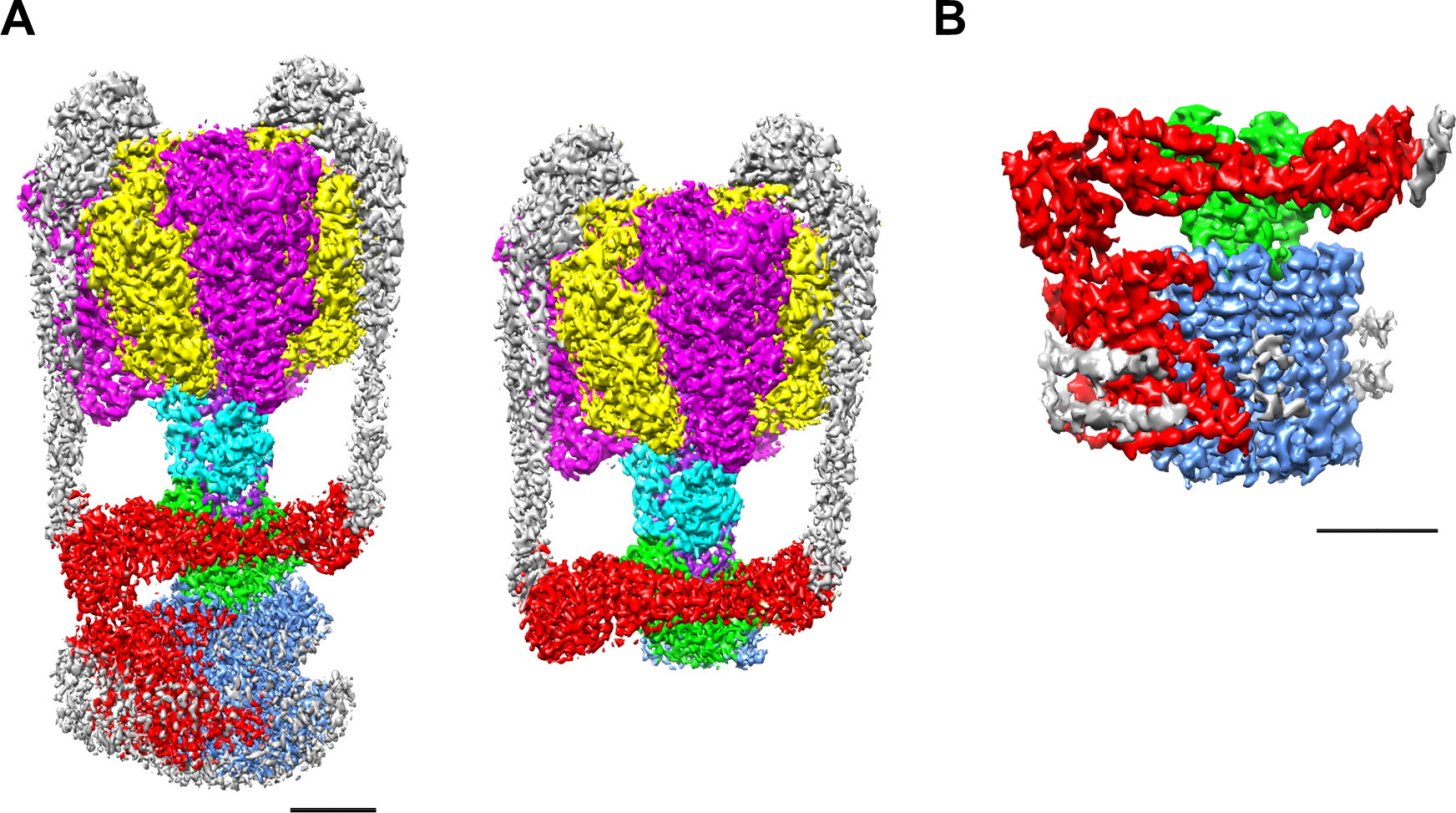
EM density map of the enzyme complex. (**A**) The *holo Tth* V/A-ATPase (left) and the focused refined map of A_3_B_3_DF*d*(EG)_2_*a*_sol_ (right). (**B**) The isolated V_o_ domain. Densities corresponding to the individual subunits are colored as follows: A, magenta; B, yellow; D, purple; F, cyan; E and G, gray; *a*, red; *d*, green; and *c*, dark blue. Scale bar = 30 Å.

We purified both the *Tth* V/A-ATPase and V_o_ domain with a His_3_-tagged *c* subunit from membranes of *T. thermophilus* cells using Ni-NTA resin. The purified complexes were reconstituted into nanodiscs composed of the membrane scaffold protein MSP1E3D1 and POPC lipids. For the *Tth* V/A-ATPase, acquisition of micrographs was carried out using the Titan Krios electron microscope equipped with a Falcon II direct electron detector. Cryo-EM micrographs of the complexes reconstituted into nanodiscs resulted in higher resolution EM maps compared to those previously reported for the LMNG solubilized preparations (Nakanishi et al., 2018). The strategy of single particle analysis for the *Tth* V/A-ATPase is summarized in Figure 2—figure supplement 1. We reconstructed the 3D structure of the *holo* complex rotational state 1 using 71,196 polished single particle images. The final structure of the state one has an overall resolution of 3.6 Å (Figure 2A). After subtracting the EM density of the membrane embedded domain from the density of the whole complex, we obtained a focused density map of A_3_B_3_D_1_F_1_*d*_1_ with two EG peripheral stalks and the soluble arm domain of the *a* subunit (*a*_sol_) at 3.5 Å resolution (Figure 2—figure supplement 5). This map allowed us to build an atomic model of A_3_B_3_D_1_F_1_ (V_1_) (Figure 2—figure supplement 6). In our map, the obvious density of ADP-Mg was observed in the closed catalytic site, but not clearly observed in the semi-closed site, in contrast to our previously reported structure of the state 1 (PDBID: 5Y5Y). The secondary ADP in the semi-closed site shows a lower occupancy due to low affinity of the semi-closed site for the nucleotide and partial flexibility of the complex (Figure 2—figure supplement 2A). In the recent cryoEM map of *Tth* V/A-ATPase (PDBID: 6QUM), clear densities, likely corresponding to ADP, were observed in the cavities of the crown-like structure formed by the six β barrel domains of A_3_B_3_ (Zhou and Sazanov, 2019). In contrast, these densities were not clearly visible in our structure (Figure 2—figure supplement 2B). This dissimilarity can presumably be explained by differences in the purification procedures; we purified the His-tagged *Tth* V/A-ATPase using a nickel column, while the authors of the previous study isolated their *Tth* V/A-ATPase without an affinity purification step.

**Figure 2—figure supplement 1.**
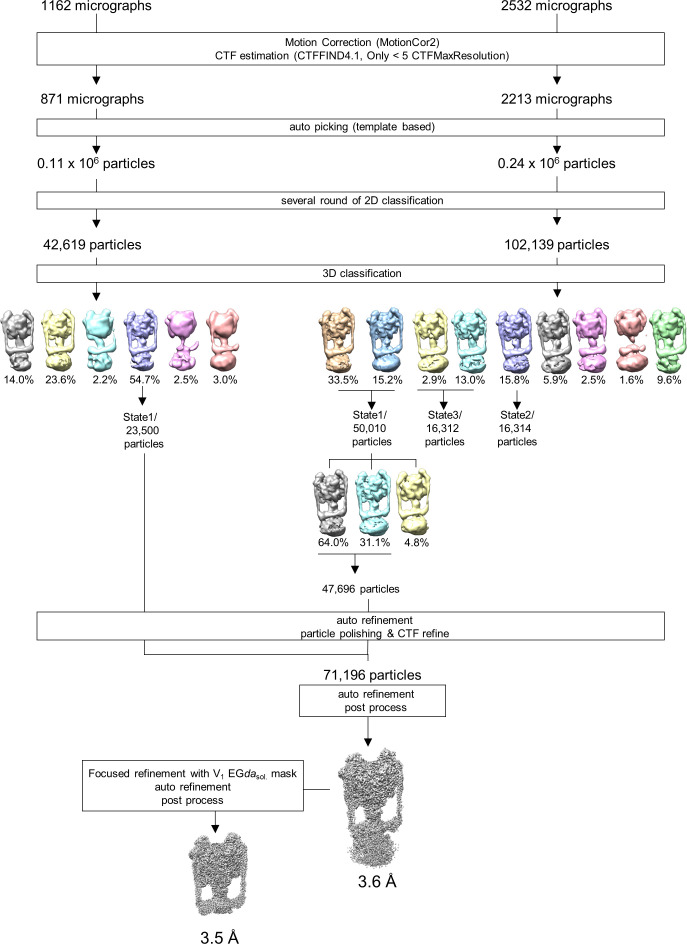
Single particle analysis of *Tth* V/A-ATPase. Flow chart of the single particle analysis process for the holo *Tth*V/A-ATPase.

**Figure 2—figure supplement 2.**
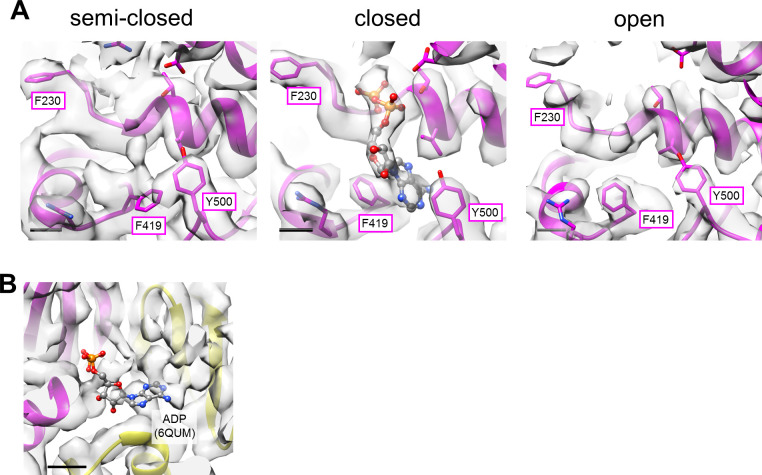
Structures of the nucleotide binding sites in the focused refined map of A_3_B_3_DF*d*(EG)_2_*a*_sol_. (**A**) The nucleotide binding site in a semi-closed AB pair (left), closed AB pair (center), and open AB pair (right). Some key residues of the A subunit, indicated in magenta box, are represented as sticks. ADP is shown as balls and sticks. Scale bar; 3 Å. (**B**) The nucleotide binding site within the crown-like structure of A_3_B_3_ proposed by Zhou and Sazanov, 2019. ADP is put in the same position as in the structure of Zhou and Sazanov (PDBID: 6QUM). Scale bar; 10 Å.

**Figure 2—figure supplement 3.**
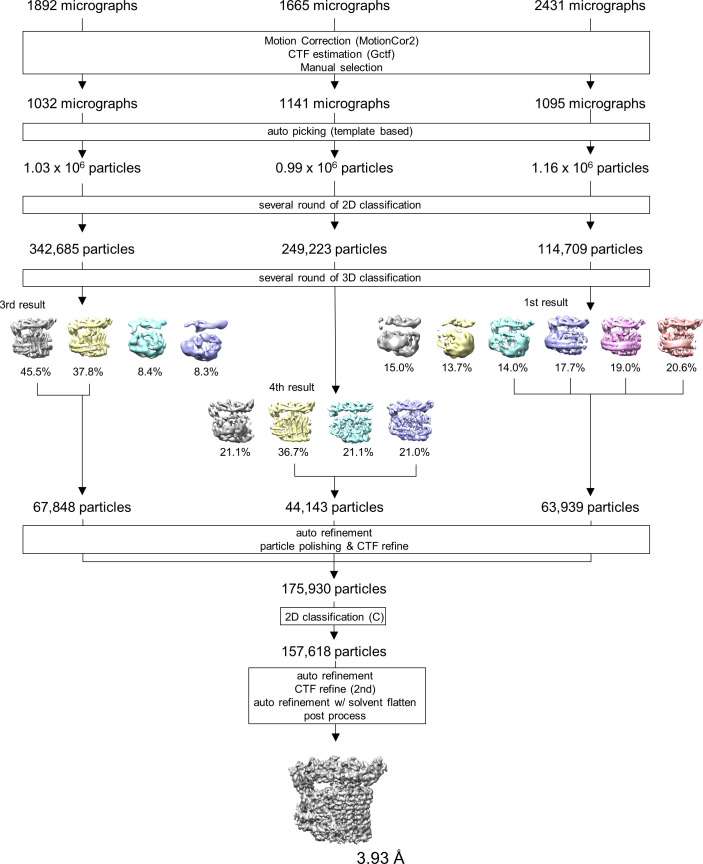
Single particle analysis of *Tth*V_o._ Flow chart of the single particle analysis process for the holo *Tth*V_o_.

**Figure 2—figure supplement 4.**
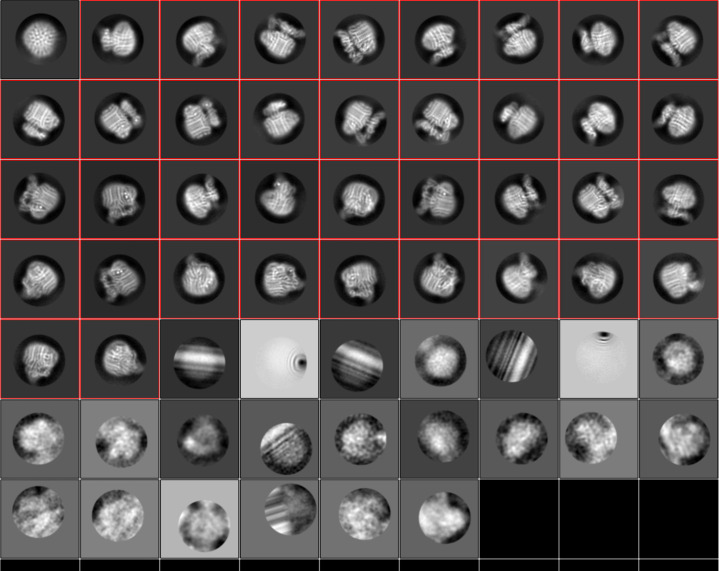
2D class averaged images of the isolated V_o_ using polished particles.

**Figure 2—figure supplement 5.**
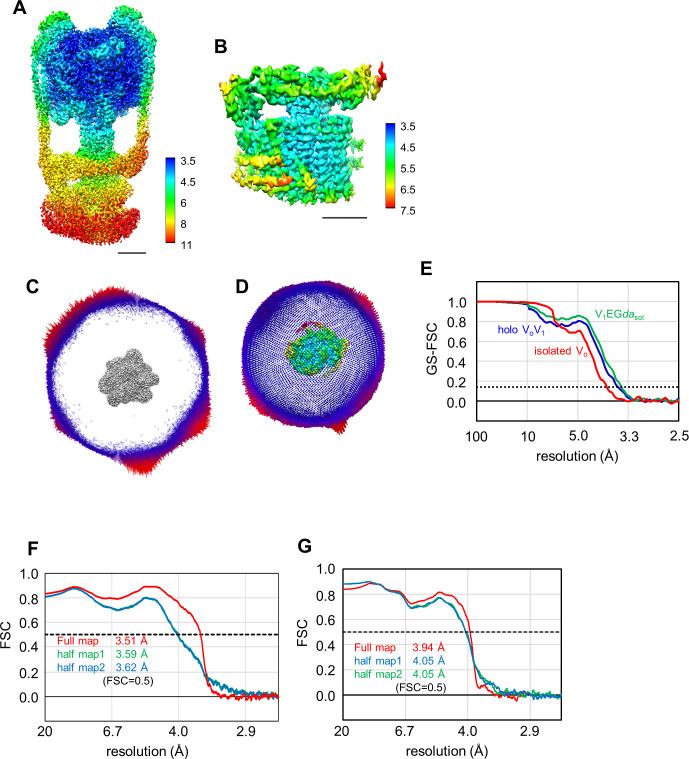
Local resolution maps of the *holo Tth*V/A-ATPase (**A**) and isolated V_o_ (**B**). Scale bar represents 50 Å for holo V/A-ATPase and 30 Å for isolated V_o_. Angular distribution of particles used for final reconstruction of the *holo Tth*V/A-ATPase (**C**), and isolated V_o_ (**D**). Fourier shell correlation (FSC) curves (**E**). Red line indicates the FSC = 0.143 criteria. FSC curves of the full map and two half maps against the built models with resolution estimated at FSC = 0.5 for V_1_EG*da*_sol_ (**F**), and isolated V_o_ (**G**).

**Figure 2—figure supplement 6.**
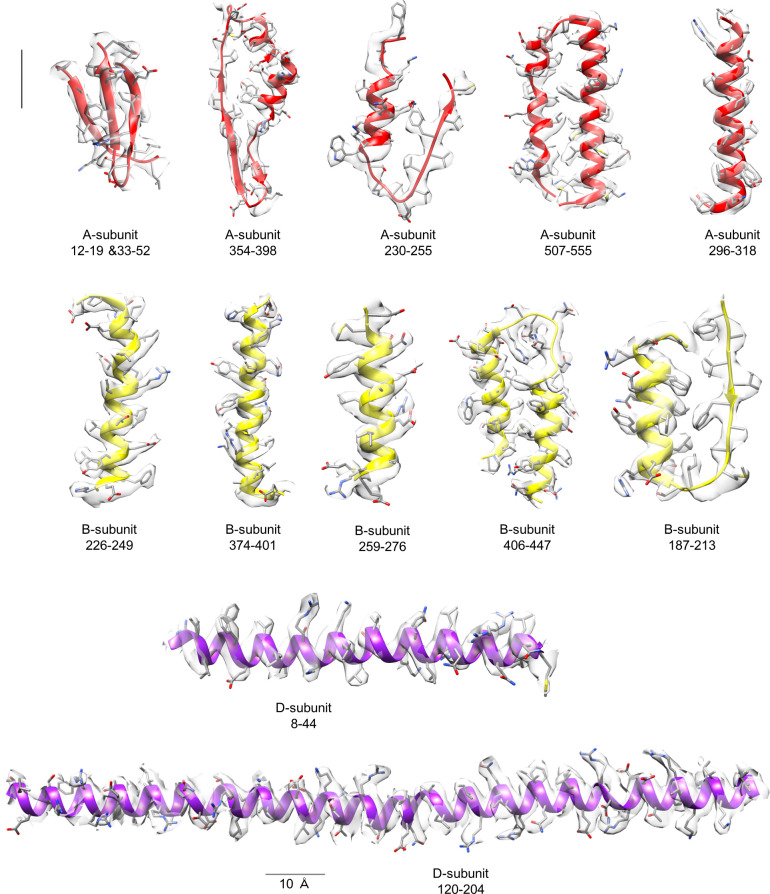
Representative densities with model fitting for each subunit of *Tth*V_1_. The built models are presented as ribbons and sticks. Residue numbers are indicated above the figures. Scale bar = 10 Å.

**Figure 2—figure supplement 7.**
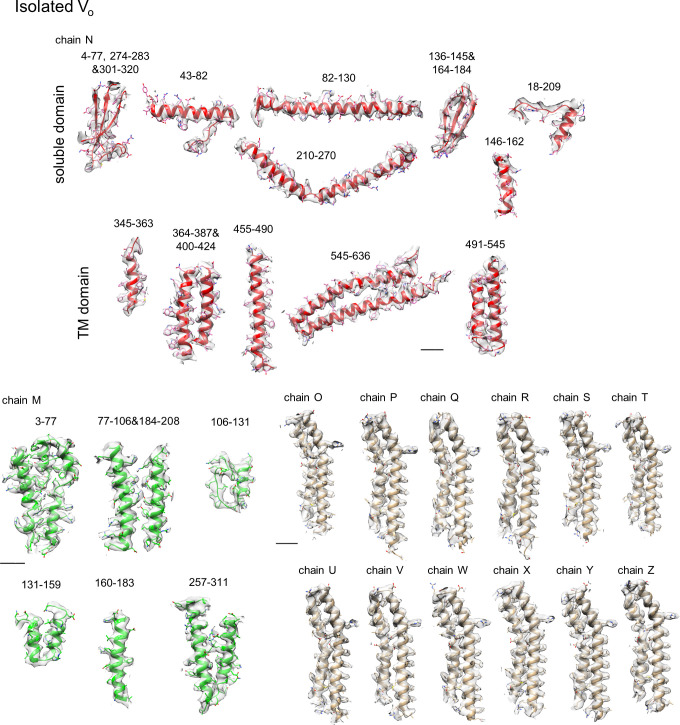
Representative densities with model fitting for each subunit of *Tth*V_o_. The built models are presented as ribbons and sticks. Residue numbers are indicated above the figures. Scale bar = 10 Å.

**Figure 3.**
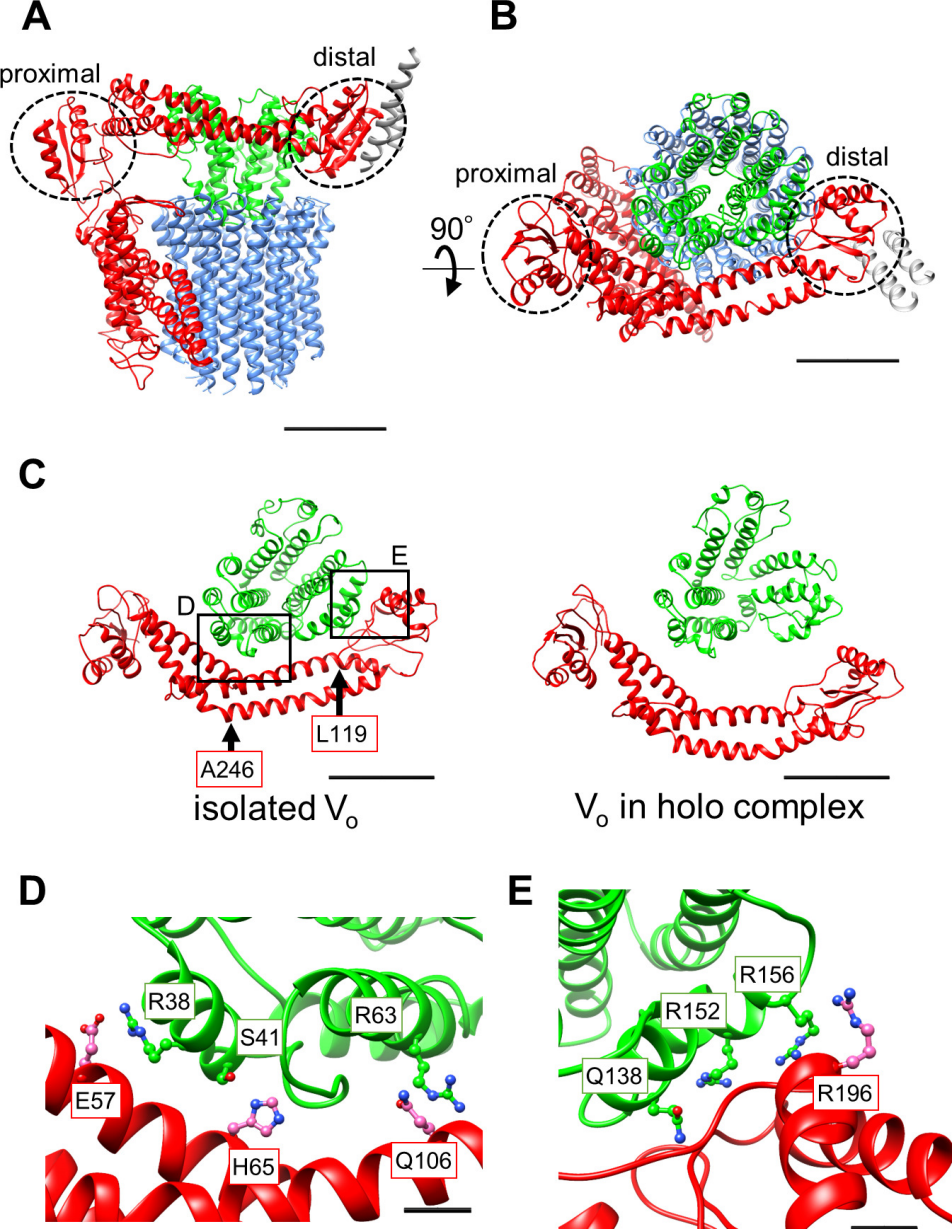
Atomic model of the isolated V_o_ domain. (**A**) Side view and (**B**) top view of *a*-, *d*-, *c*-, and EG subunits colored as in Figure 2, respectively. Scale bar represents 30 Å. The proximal and distal subdomains of the *a*-subunit are circled by dotted lines. (**C**) Comparison of the relative positions of *a*_sol_ (red) and the d subunit (green) in the isolated V_o_ domain (left) and the V_o_ domain in the *holo* complex (right). Arrows indicate the kinking and twisting points in the *a*_sol_ region of the isolated V_o._ Scale bar represents 30 Å. (**D**) and (**E**) Specific interactions between the *a*_sol_ region and *d* subunit at the proximal (**D**) and distal (**E**) regions. The regions are specified by black squares in C. Scale bar = 5 Å.

The purified V_o_ domain reconstituted into nanodiscs was subjected to single particle analysis using a cryoEM (CRYOARM200, JEOL) equipped with a K2 summit electron direct detector in electron counting mode. The strategy of single particle analysis for *Tth*V_o_ is summarized in Figure 2—figure supplement 3. The 2D class averages disclosed the isolated V_o_ domain with clearly visible transmembrane helices and a hydrophilic domain extending above the integral membrane region (Figure 2—figure supplement 4). The scaffold proteins and lipids of the nanodiscs surrounding the membrane domain of the isolated V_o_ were clearly visible. Subsequent 3D classification of the observed V_o_ states revealed only one major class, indicating that the isolated V_o_ is structurally homogenous, in contrast to the *Tth* V/A-ATPase, which was clearly visible in three different rotational states (Nakanishi et al., 2018). Our 3D reconstruction map of the isolated V_o_ complex was obtained with an overall resolution of 3.9 Å (Figure 2—figure supplement 5). The final map shows clear density for protein components of V_o_, including subunit *a*, subunit *d* and the *c*_12_ ring, but the EM density for both EG stalks, which attach to the *a*_sol_ region, is weak indicating disorder or flexibility in these regions (Figure 2B). In this structure, a C-terminal region of the EG stalk on the distal side is visible. With the exception of these two EG stalks, side-chain densities are detectable for most of the proteins in the complex, allowing construction of a de novo atomic model using Phenix and Coot software (Figure 3A,B, Figure 2—figure supplement 7). The map contains an apparent density inside the *c*_12_ rotor ring, likely corresponding to the phospholipids capping the hole of the ring (Figure 3—figure supplement 1A). A further apparent density was identified in the cavity between the *a* subunit and *c*_12_ ring on the upper periplasmic side (Figure 3—figure supplement 1B). This density may also correspond to phospholipids, and we suppose that it functions to plug the cavity between the *a* subunit and *c*_12_ ring, preventing proton leak from the periplasmic proton pathway. Similar densities corresponding to phospholipids were also observed in the recently published cryoEM density map of the *holo* complex (Zhou and Sazanov, 2019). Notably, the diameter of the *c*_12_ rotor ring in the isolated V_o_ is slightly smaller than that in the *Tth* V/A-ATPase (Figure 3—figure supplement 2). It is likely that penetration of the short helix of the subunit D into the subunit cavity of subunit *d* enlarges the diameter of the *c*_12_ rotor ring in the *Tth* V/A-ATPase.

**Figure 3—figure supplement 1.**
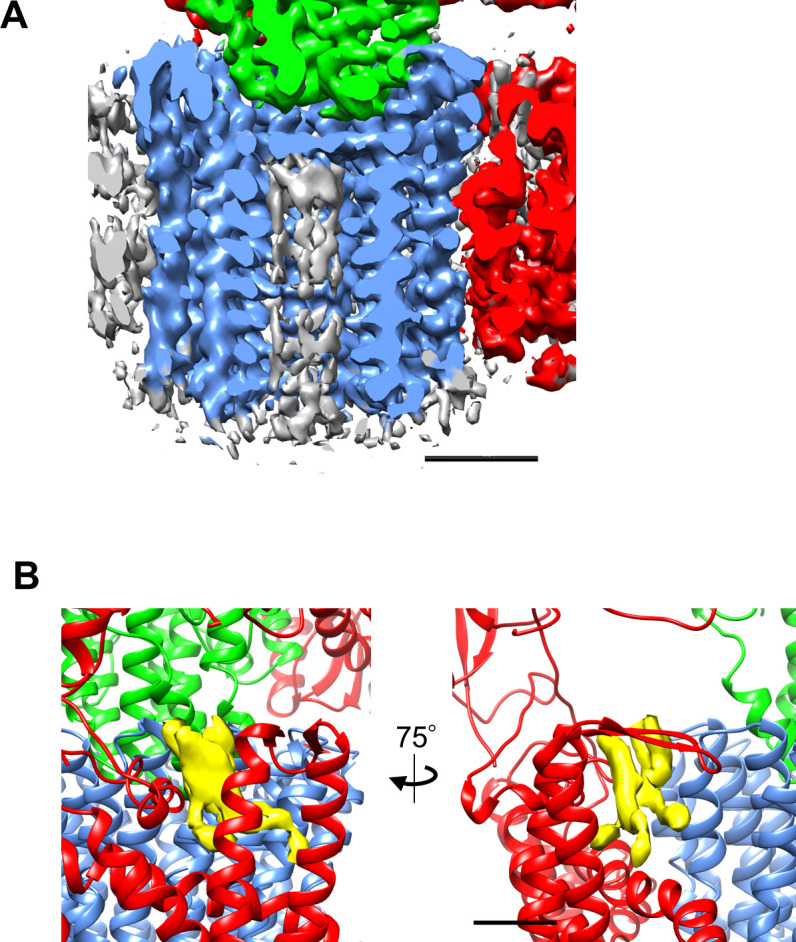
The density corresponding to lipids in *Tth* V_o_. (**A**) The cross section of the *c*-ring region of the V_o_ density map perpendicular to the cell membrane. The density is colored according to the individual subunits; *a* (red), *d* (green), and *c* (dark blue). Density corresponding to lipid was observed inside the *c*-ring (grey). (**B**) Density corresponding to lipid was observed between the *a*-subunit and *c*-ring (yellow).

**Figure 3—figure supplement 2.**
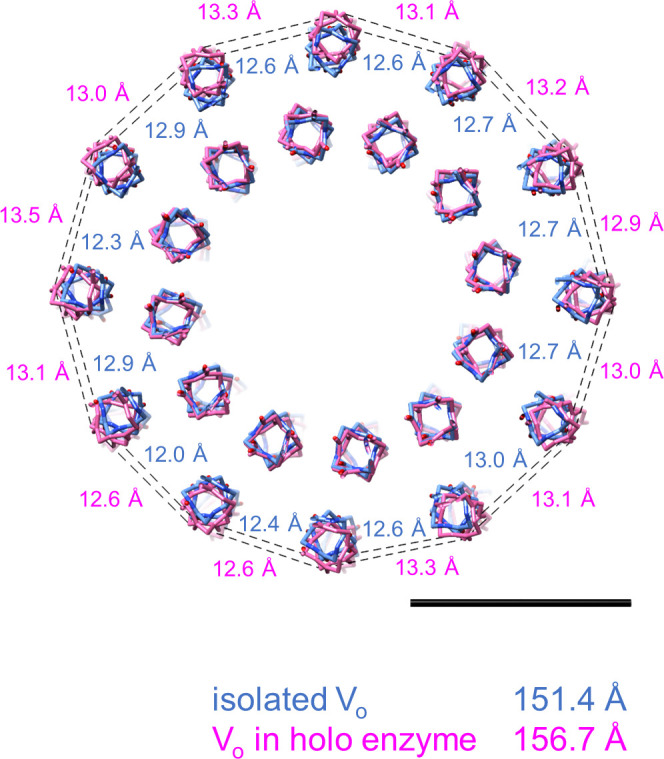
Comparison of circumferences of the rotor *c*-ring in the isolated V_o_ and *holo*-complex. The dotted lines show the distance between Cα of L71 in adjacent subunits. Scale bar = 20 Å.

### Structure comparison of the isolated and complexed V_o_ domains

A comparison of structures determined for the isolated V_o_ domain with that in the *holo* complex revealed a high degree of similarity in the membrane embedded region. However, there were significant differences in the *a* subunit. The basic structure of the *Tth* V_o_ *a* subunit is almost identical to the eukaryotic counterpart, comprising a soluble arm domain (*a*_sol_) and a C-terminal hydrophobic domain responsible for proton translocation via rotation of the *c*_12_ ring. The *a*_sol_ region contains two globular α/β folding subdomains responsible for binding of both the proximal and distal EG stalks (Figure 3A and B). Both globular subdomains are connected by a hydrophilic coiled coil with a bent conformation.

In contrast to the V_o_ structure in the *holo* complex, the *a*_sol_ region in the isolated V_o_ is located in close proximity to the *d* subunit as a result of kinking and twisting of the coiled coil at residues *a*/L119 and *a*/A246 (Figure 3C, indicated by the arrows). In this structure, several interactions between the *d* subunit and *a*_sol_ residues can be observed (Figure 3D). At the proximal site, three amino acid residues, *a*/E57, *a*/H65, and *a*/Q106, form salt bridges or hydrogen bonds with residues *d*/R38, *d*/S41, and *d*/R63 of the *d* subunit, respectively. Our structure also reveals clearly connected densities between the distal subdomain of the *a*_sol_ region and *d* subunit (Figure 3E). Four side chains, *d*/Q138, *d*/R152, *d*/R156, and *a*/R196 apparently form hydrogen bonds with the oxygen atoms in the main chain of *a*/E201, *a*/L144, *a*/A197, and d/R156, respectively. With the exception of the interaction between *a*/E57 and *d*/R38 in the proximal site, these interactions are broken by the dynamic movement of *a*_sol_ and conformational changes of the *d* subunit in the V_o_ moiety of the *holo Tth* V/A-ATPase. The conformational changes induced by binding of V_1_ (A_3_B_3_DF) to V_o_ are described in a separate section below.

### Structure of the membrane embedded region of the isolated V_o_ domain

**Figure 4.**
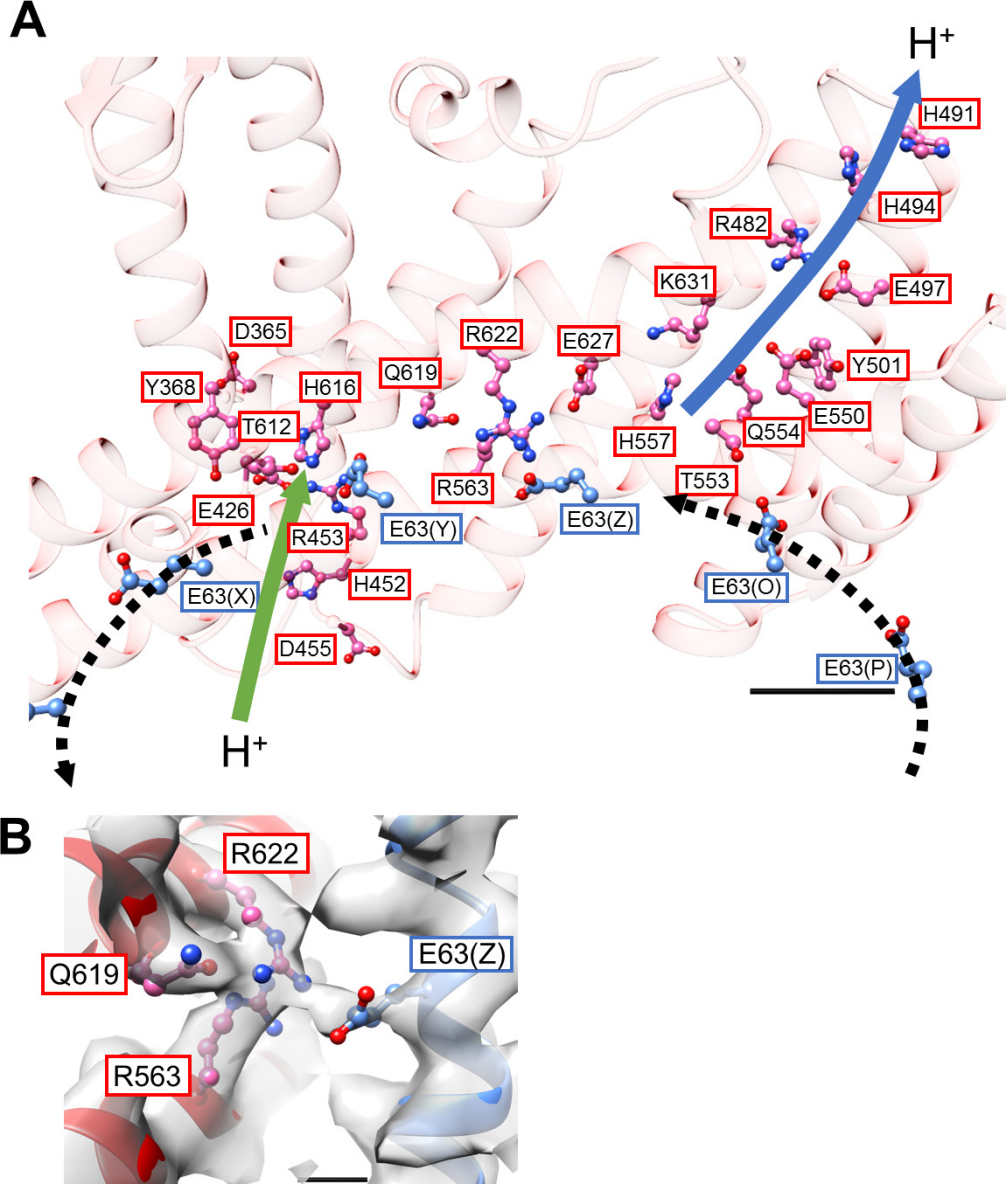
Structure of the hydrophobic domain of isolated V_o_. (**A**) The half-channels for proton translocation on both the cytoplasmic and periplasmic sides of the isolated V_o_ domain. Residues lining the pathways are represented as balls and sticks. Residues from the *a*-subunit and *c*-subunit are indicated in red and blue boxes, respectively. Proton flow, as it would occur in the case of ATP synthesis, is represented by arrows. The solid arrows indicate proton flow from the periplasmic side to the *c*-subunit (green), and from the *c*-subunit to the cytoplasmic side (blue). The dotted black arrows indicate proton movement due to rotation of the *c*_12_-ring. Scale bar = 10 Å. (**B**) The salt bridge between *a*/Arg563, Arg622, Gln619 and *c*/Glu63. Scale bar = 3 Å.

Our previous low-resolution structure of the *Tth* V/A-ATPase suggested the involvement of half-channels in proton translocation on both the cytoplasmic and periplasmic sides of the V_o_ domain (Nakanishi et al., 2018). The atomic model of V_o_ presented here reveals details of the half-channels formed by the membrane-embedded C-terminal region of the *a* subunit (*a*_CT_) and its interface with the *c*_12_ ring. The *a*_CT_ region contains eight membrane-embedded helices, MH1 to MH8. MH7 and MH8 are the highly-tilted membrane-embedded helices characteristic of rotary ATPases. The cytoplasmic hydrophilic cavity is formed by the cytoplasmic side of MH4, MH5, MH7, and MH8, and the *c* subunit/chain Z. The cavity is lined by the polar residues, *a*/R482, *a*/H491, *a*/H494, *a*/E497, *a*/Y501, *a*/E550, *a*/Q554, *a*/T553, *a*/H557, and *c*(Z)/Thr54 (Figure 4A), which make up the cytoplasmic half-channel. The periplasmic sides of MH1, MH2, MH7, and MH8 form the periplasmic hydrophilic cavity, lined with *a*/D365, *a*/Y368, *a*/E426, *a*/H452, *a*/R453, *a*/D455, and *c*(Y)/E63. The two hydrophilic channels are separated by a salt bridge formed between *c*(Z)/63Glu, a residue critical for proton translocation, and *a*/Arg563, *a*/Arg622, *a*/Gln619 of MH7 (Figure 4B). This salt bridge is conserved in both eukaryotic and prokaryotic V_o_ (Mazhab-Jafari et al., 2016; Kishikawa and Yokoyama, 2012). Of note, a salt bridge forms between a single arginine residue and a single glutamic (or aspartic) acid residue in F_o_ (Kühlbrandt, 2019; Murphy et al., 2019; Guo et al., 2019). Similar to the two-channel model described for other rotary ATPases (Srivastava et al., 2018; Hahn et al., 2018), the two arginine residues on the MH7 and MH8 play an important role in protonation and deprotonation of the carboxy groups on the *c*_12_ ring, with the resulting rotation of *dc*_12_ driven by proton translocation from the periplasmic to cytoplasmic side (Guo and Rubinstein, 2018; Hahn et al., 2018; Pogoryelov et al., 2010). Notably, in addition to the rigid salt bridge formed between the two *a*/Arg residues, *a*/Gln and *c*/Glu, further interactions between *a*_ct_ and the *c*_12_ ring are observed. Furthermore, *a*/Asp392 and Leu393 -*c*(Y)/Arg49 in the loop region of the *c* subunit (Figure 4—figure supplement 1A), and the periplasmic sides of MH5 and MH6 are in close proximity to the C-terminal end of the *c* subunit (Figure 4—figure supplement 1B). These interactions are observed in the V_o_ moiety of the recently published *holo* complex structure (Zhou and Sazanov, 2019). Overall, our V_o_ structure is largely identical to the V_o_ moiety observed in the *holo* complex with the exception of some alterations in the hydrophilic domain (Zhou and Sazanov, 2019).

**Figure 4—figure supplement 1.**
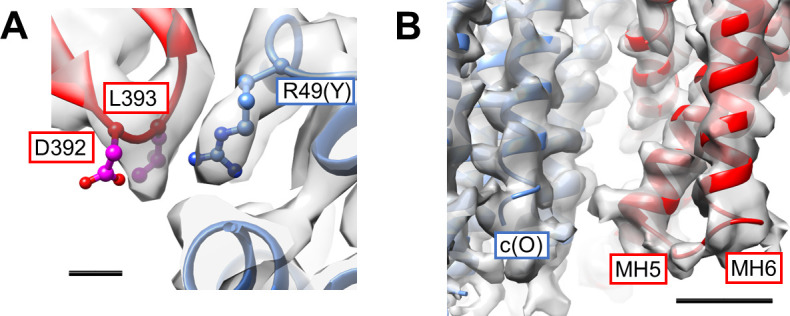
Interactions between the *a*-subunit and c_12_-ring. (**A**) The cytosolic loop between MH2 and MH3 close to *c*/Arg49. Scale bar = 3 Å. (**B**) The periplasmic side of MH5 and MH6 in close proximity to the C-terminal end of the c subunit. Scale bar = 10 Å.

### Voltage threshold for proton conductance activity of the isolated V_o_ domain

**Figure 5.**
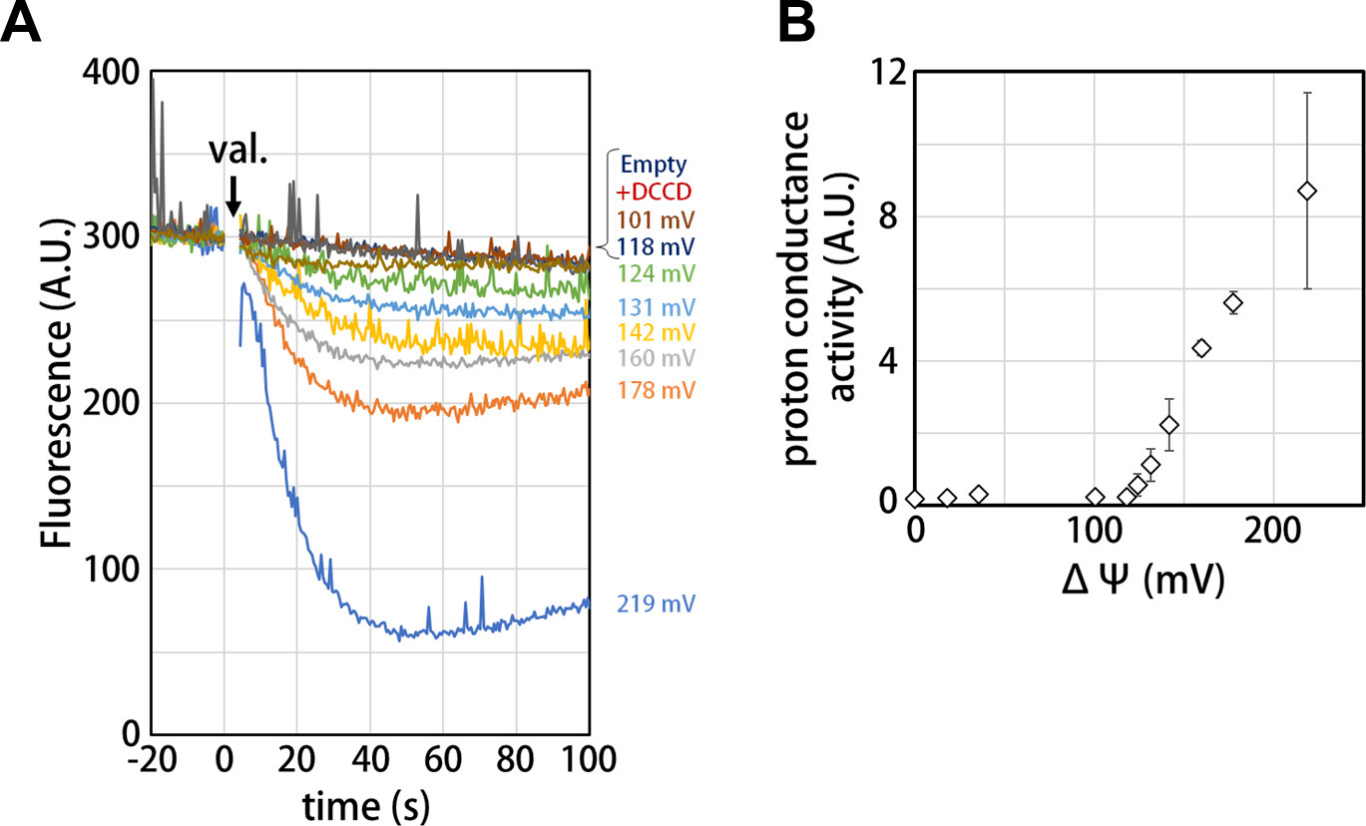
Proton conductance through the isolated V_o_ domain. (**A**) Changes of ACMA fluorescence due to pH changes inside the V_o_ proteo-liposomes. Values of the membrane potential (ΔΨ) were estimated using the Nernst equation, ΔΨ = *RF*/*zF* ln[KCl]_o_/[KCl]_i_, as described in the Materials and methods section. (**B**) The voltage threshold of proton conductance through the V_o_ domain (mean ± SD, n = 3).

Our structure of the isolated V_o_ domain suggests that the rotation of the *c*_12_ rotor ring relative to the stator is mechanically hindered by a defined interaction between the *a*_sol_ region and the *d* subunit. Previous studies have shown that isolated yeast V_o_ is impermeable to protons (Couoh-Cardel et al., 2015; Qi and Forgac, 2008), but it was unclear whether proton conductance is also inhibited in the isolated *Tth* V_o_ domain. To investigate proton conductance through the isolated *Tth* V_o_, we reconstituted this domain into liposomes energized with a *Δψ* generated through a potassium ion (K^+^)/valinomycin diffusion potential. The pH change in the liposomes was monitored with 9-Amino-6-Chloro-2-Methoxyacridine (ACMA); the emission traces at 510 nm excited at 460 nm were recorded (Figure 5). The membrane potential was modulated by varying the external K^+^ concentration according to the Nernst equation. As shown in Figure 5B, the isolated V_o_ domain displays no proton conductance when the membrane potential is lower than 120 mV, defining a voltage threshold. The proton conductance through the V_o_ increases proportionally with the membrane potential when the membrane potential exceeds 130 mV (Figure 5B). The reported membrane potential in bacterial cells varies from −75 to −220 mV depending on growth environment and method of quantification (Lo et al., 2007; Bot and Prodan, 2010). Although the membrane potential of *T. thermophilus* under physiological conditions is unknown, we reported previously that the *Tth* V/A-ATPase is capable of ATP synthesis when the membrane potential exceeds −110 mV (Toei et al., 2007). Thus, proton impermeability of the isolated *Tth* V_o_ observed at potentials less than −120 mV may function to maintain *pmf* for ATP synthesis, when *Tth* V_o_ exists solely on the cell membrane. In contrast to the V_o_ domain, several experiments have indicated that proton conductance through the bacterial F_o_ domain is not sensitive to any specific threshold in membrane potential (Wiedenmann et al., 2008), whereas bacterial F_o_F_1_ is sensitive to a membrane potential threshold, likely dependent on the interaction between F_o_ and F_1_ (Feniouk et al., 2004). In addition, proton conductance through the F_o_ domain increases linearly with increasing *Δψ* loaded on the F_o_ liposome. These results indicate that there are no or few interactions between the *a* subunit and *c*-ring to hinder *c*-ring rotation in F_o_. Together, the observed results suggest that *a*_sol_ of the *a* subunit and the *d* subunit, absent from F_o_ and validated structures of the V type ATPases, can be one of the keys for mechanical inhibition of proton conductance through V_o_.

## Discussion

**Figure 6.**
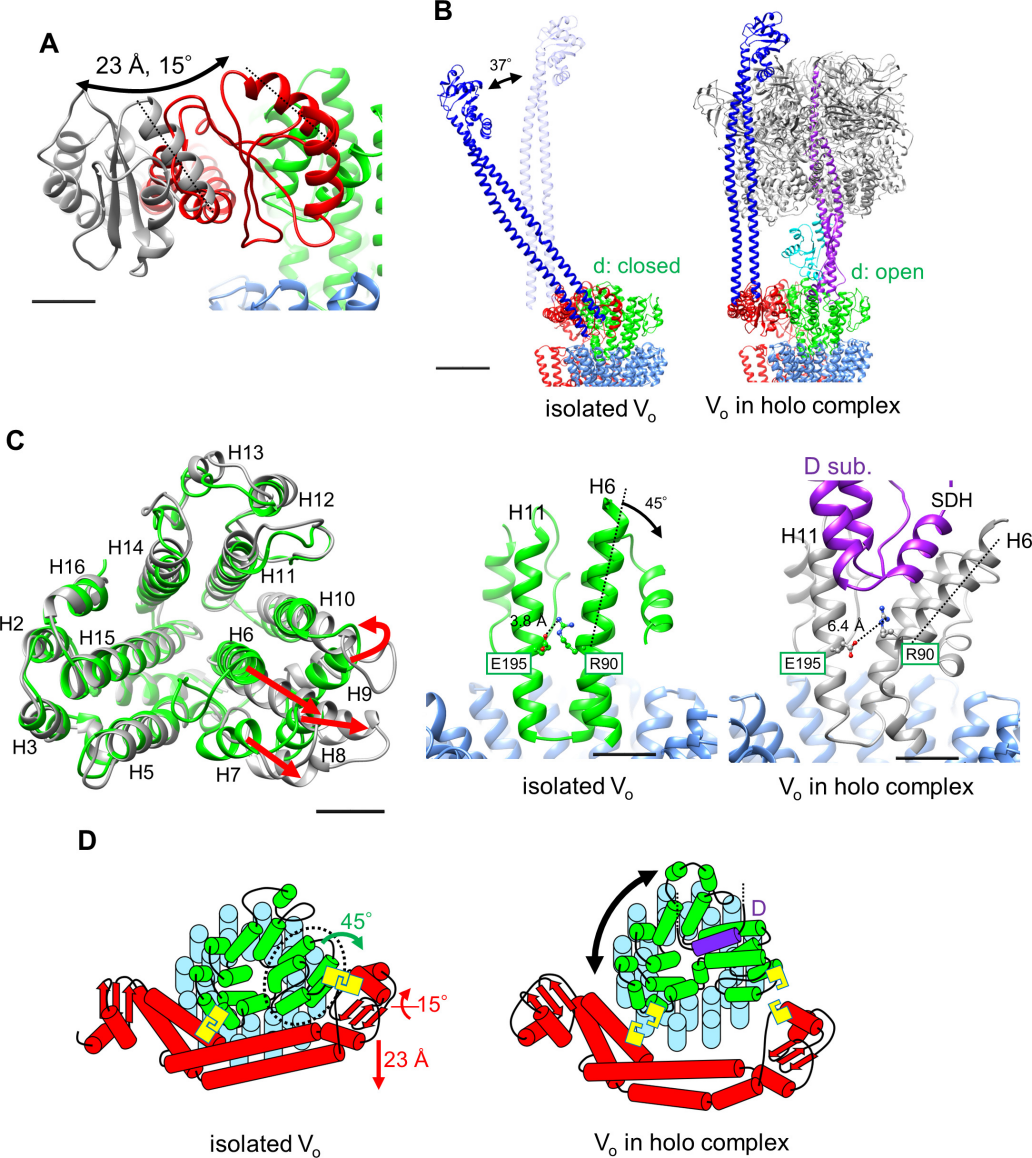
Conformational changes occurring in both the *d*- and *a*_sol_ subunits as a result of V_1_ to V_o_ binding. (**A**) Structural changes in the *d* subunit caused by insertion of the screw driver helix (SDH). A top view of the *d*-subunit is shown in the left panel. The *d*-subunit from the isolated V_o_ domain and the *holo* enzyme are colored in green and grey, respectively. Red arrows indicate movements of helices 6–9 (H6–H9). The key helices, H6 and H11, of the *d*-subunit in the isolated V_o_ domain and *holo* complex are shown in panel A, center and right. The H6 helix bends 45° as a result of interaction between the d-subunit and SDH. (**B**) Structural changes in the distal subdomain of *a*_sol_. Upon the pivoting movement of *a*_sol_ on the proximal subdomain, the distal subdomain swings 25 Å and twists 15° between the isolated V_o_ (red) and the *holo* complex (gray). (**C**) The EG structure in the distal subdomain of *a*_sol_ (EG_d_) in the isolated V_o_ domain (left) and in the *holo* complex (right). (**D**) A schematic representation of mechanical inhibition of V_o_ induced by dissociation of V_1_. In the isolated V_o_ domain, rotation of the central rotor is inhibited by interactions between the *d*-subunit and *a*_sol_ (yellow box, Figure 3D, E).

The structure of the isolated *Tth* V_o_ obtained clearly shows a different conformation from the V_o_ moiety in the holo-complex. From structural comparison between isolated V_o_ and the *holo* complex, it can be suggested that structural changes in isolated V_o_ observed in two subunits were most likely induced by dissociation of the V_1_ domain from V_o_. In the isolated V_o_ domain, the *d* subunit adopts the closed form in which three side chains of the *d* subunit are able to interact with the distal subdomain of *a*_sol_ (Figure 3E). Once the short helix of the D subunit, an axis subunit of the V_1_ domain, inserts into the cavity of the *d* subunit, the interaction between H6 and H11 via *d*/R90 and *d*/E195 is broken (Figure 6A and Video 1), resulting in the *d* subunit adopting an open form, with side chains orientated away from the distal subdomain of *a*_sol_.

**Figure 6—figure supplement 1.**
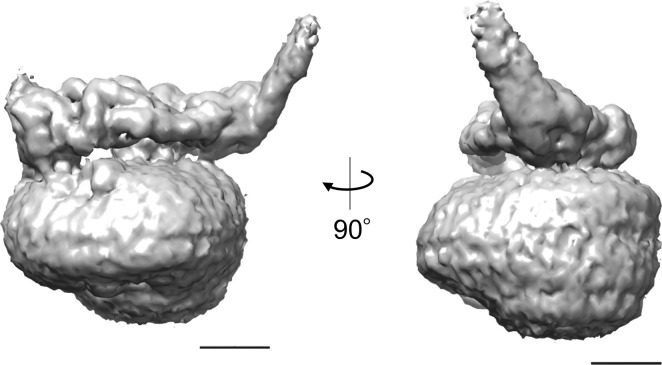
Low resolution electron density map of isolated V_o_. The density map clearly indicates that the EG subunit at the distal subdomain of *a*_sol_ tilts away from the V_1_ side. Scale bar = 20 Å.

**Figure 6—figure supplement 2.**
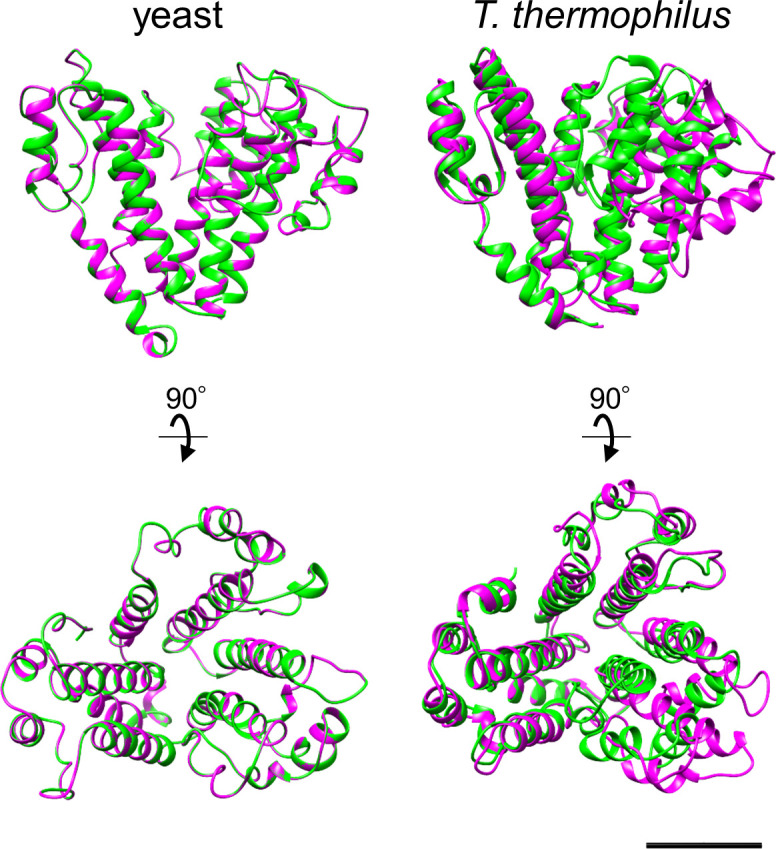
Conformational changes in the *d* subunit induced by association of V_1_ with V_o_ in yeast V-ATPase (left) and in *Tth*V/A-ATPase (right). Structure of the *d* subunit in isolated V_o_ is shown in green and structure of the *d* subunit in the *holo* complex is shown in magenta. PDBID: yeast V_o_ (6O7U), yeast V-ATPase (6O7V), *T. thermophilus* V/A-ATPase (6QUM). Scale bar = 20 Å.

**Video 1.** Conformational changes of the *d*-subunit between isolated V_o_ and V_o_ in holo-enzyme. A morphed movie focuses on the conformational changes of the *d*-subunit between isolated V_o_ and V_o_ in the holo-enzyme. *a*-, *d*-, *c*- and *d*-subunits are colored in red, green, dark blue, and orange, respectively. *d*/R90 and *d*/E195 are represented as balls and sticks. The density map of isolated V_o_ is shown as a semi-transparent surface.

Another contributing factor is the dynamic motion of the *a*_sol_ region induced by binding the distal EG stalk to the top of the A_3_B_3_ from the V_1_ domain. In the isolated V_o_, the N-terminal region of the EG stalk bound to the distal subdomain of *a*_sol_ is at a much steeper angle relative to the horizontal coiled coil structure of *a*_sol_ than that in the *holo* enzyme (Figure 6B,C and Figure 6—figure supplement 1). This finding suggests that the stalk region also adopts a steep angle, although the stalk and head domain of EG are disordered in the resolved structure and thus not visible in the density map (Figure 2B). Once the C-terminal globular domain of the distal EG stalk binds onto the top of A_3_B_3_, the angled distal EG adopts a vertical standing form, resulting in both twisting and kinking of the coiled coil of the hydrophilic arm and the distal globular subdomain of the *a* subunit (Figure 6C, Video 2). These dynamic motions of *a*_sol_ induce disruption of specific interactions between *a*_sol_ and the *d* subunit.

**Video 2.** Conformational changes of a hydrophilic arm of the *a*-subunit linked to movement of the EG subunits. A morphed movie focusing on the conformational changes of a hydrophilic arm of the *a*-subunit between isolated V_o_ and V_o_ in the holo-enzyme. EG*_p_* and EG*_d_* indicate proximal and distal EG subunits. The position of EG*_d_* was determined from the low-resolution density map (Figure 6—figure supplement 1). The hydrophilic arm of the *a*-subunit associated with binding EG*_d_* to one of the B subunits from V_1_, is forced to swing away from the *d*-subunit, resulting in disruption of the specific interaction between the subunits.

The isolated yeast V_o_ domain also adopts a conformation where the *a*_sol_ region is in close proximity to the *d* subunit, resulting in rigid interaction between the stator and rotor that is advantageous for inhibition of proton conductance (Roh et al., 2018; Mazhab-Jafari et al., 2016). Although an atomic model of the yeast *holo* V-ATPase has yet to be determined, a poly alanine model of the yeast V-ATPase shows that the *a*_sol_ region is some distance away from the *d* subunit in the V_o_ moiety (Zhao et al., 2015). In addition, a recently reported structure of the mammal V-ATPase clearly shows that *a*_sol_ is at a distance where it cannot interact with the *d* subunit (Abbas et al., 2020). This structure suggests that a similar conformational change in V_o_ is induced by binding of the V_1_ domain in the yeast V-ATPase, as described by Oot and Wilkins previously (Oot and Wilkens, 2012). Notably, the *d* subunit in the yeast enzyme differ in conformation between the isolated V_o_ domain and *holo* enzyme, in contrast to the *Tth* enzyme, where the *d* subunit is in the closed form in the isolated V_o_ domain (Figure 6—figure supplement 2; Vasanthakumar et al., 2019). The *d* subunit from the mammalian *holo* V-ATPase adopts a more open conformation than the yeast *d* subunit from the isolated V_o_ complex, as seen in the *holo Tth* V/A-ATPase (Abbas et al., 2020). In addition, Abbas et al. suggest that the *d* subunit from the yeast *holo* V-ATPase is also more open compare to that of the yeast isolated V_o_ (Abbas et al., 2020). These results indicate that the *d* subunit in the mammalian and yeast V-ATPase also exhibits a conformational change between isolated V_o_ and *holo* enzyme.

However, Qi et al. reported that yeast V_o_ was impermeable to proton even in the absence of interactions between the *a*-subunit and *d*-subunit (Couoh-Cardel et al., 2015; Qi and Forgac, 2008). These findings suggest that the interactions between *a*_sol_ and *d*-subunit are not the only mechanism by which proton permeability is inhibited. In fact, salt bridges between the arginine residues (*a*/R563, R622 in *Tth* V_o_, *a*/R735, 799 in yeast V_o_) and the glutamate residue (*c*/E63 in *Tth* V_o_, *c*/E108 in yeast V_o_) (Roh et al., 2018; Mazhab-Jafari et al., 2016; Vasanthakumar et al., 2019) are identified in isolated V_o_ from both *T. thermophilus* and yeast V_o_. These salt bridges between the stator *a* subunit and the rotor *c*-ring inhibit proton permeability by hindering *c*-ring rotation (Mazhab-Jafari et al., 2016). It is still controversial whether the formation of this salt bridge represents a *bona fide* process of proton translocation that links deprotonation and re-protonation of glutamate residues in the *c* subunits. (Kühlbrandt, 2019; Roh et al., 2018; Zhou and Sazanov, 2019; Abbas et al., 2020; Krah et al., 2019; Pierson et al., 2018; Symersky et al., 2012). Undoubtedly, the salt bridge must be broken by both rotation of the *c*-ring driven by *pmf* and ATP hydrolysis in V_1_ in order to perform the functions of ATP synthesis or proton pumping (Figure 5; Roh et al., 2018; Vasanthakumar et al., 2019; Abbas et al., 2020).

As described above, eukaryotic and prokaryotic V/A-ATPases appear to share a similar mechanism of conformational change at the V_o_ moiety, advantageous for preventing proton leakage from cells or acidic vesicles. Nevertheless, there exist some interactions unique to *Tth* V_o_, as described in this paper (Figure 4—figure supplement 1), and to yeast V_o_, as reported by previous studies (Roh et al., 2018). This suggests that the auto-inhibition mechanisms of V_o_ have been conserved during evolution of V type ATPases.

In the isolated yeast V_o_, the luminal half-channel, which releases translocated protons to the lumen of acidic vesicles is closed and it is assumed to open transiently during catalysis (Mazhab-Jafari et al., 2016; Krah et al., 2019). In the case of *Tth* V/A-ATPase, both sides of the half-channel are open (Zhou and Sazanov, 2019). The membrane domain of the *a* subunit from the isolated *Tth* V_o_ is largely identical to that of the *holo* enzyme (r. m. s. d. = 0.82 Å for A327-E637 of the *a* subunit), thus the half-channels are likely also open in *Tth* V_o_ as observed in the *holo* enzyme. This indicates that *Tth*V_o_ is more proton permeable than yeast V_o_. This difference might be a consequence of the differences between the protein acting as an ATP synthase (*Tth* enzyme) and ATP driven proton pump (yeast enzyme), as previously suggested (Krah et al., 2019). Further studies, such as computational MD simulation, are required to assess the extent of contribution of each interaction to the auto-inhibition mechanism of *Tth* V_o_.

Our structure of the isolated V_o_ domain further reveals the mechanism of mechanical inhibition of rotation of the *dc*_12_ rotor complex due to strong interactions between *a*_sol_ and the *d* subunit, regions unique to V-ATPases. These interactions stabilize the isolated V_o_ domain and protect against loss of the *d*-subunit in the absence of the rotor-stator interactions mediated by V_1_ in the *holo* enzyme (Ediger et al., 2009). This stabilization of V_o_ is likely to be a key factor for both assembly of *holo* V-type ATPase complexes and regulation of the eukaryotic V-ATPase via dissociation of V_1_ from V_o_.

## Materials and methods

**Key resources table:** 

Reagent type (species) or resource	Designation	Source or reference	Identifiers	Additional information
Strain, strain background *T. thermophilus*	HB8	Tamakoshi et al., 1997		
Chemical compound, drug	14:0 PC (DMPC)	Avanti polar lipid	850345	
Chemical compound, drug	16:0-18:1 PC (POPC)	Avanti polar lipid	850457	
Chemical compound, drug	n-Dodecyl-beta-D-maltopyranoside	cosmo bio	D-1304	
Chemical compound, drug	Biobeads SM-2	bio-rad	1523920	
Chemical compound, drug	L-α-Phosphatidylcholine from soybean, Type II-S	Merck	P5638	
Chemical compound, drug	n-octyl-β-D-glucoside	sigma aldrich	850511P	
Chemical compound, drug	9-Amino-6-Chloro-2-Methoxyacridine	Thermo Fisher	A1324	
Chemical compound, drug	Carbonyl cyanide 4-(trifluoromethoxy)phenylhydrazone	sigma aldrich	C2920	
Chemical compound, drug	Valinomycin	sigma aldrich	V0627	
Software, algorithm	RELION	Zivanov et al., 2018	RRID:SCR_016274	
Software, algorithm	MotionCor2	Zheng et al., 2017	RRID:SCR_016499	
Software, algorithm	Gctf	Zhang, 2016	RRID:SCR_016500	
Software, algorithm	COOT	http://www2.mrc-lmb.cam.ac.uk/personal/pemsley/coot/	RRID:SCR_014222	
Software, algorithm	Phenix	https://www.phenix-online.org/	RRID:SCR_014224	
Software, algorithm	MolProbity	http://molprobity.biochem.duke.edu	RRID:SCR_014226	

### Protein preparation

T. thermophilus V/A-ATPase was expressed with a His_3_ tag on the C-terminus of the c-subunit using a modified operon generated by the integration vector system (Tamakoshi et al., 1997). Purification of His-tagged V_o_ was carried out as described previously (Nakanishi et al., 2015). Briefly, membranes of *T. thermophilus* were suspended in a buffer containing 10% Triton X-100 and sonicated to solubilize membrane proteins. After ultracentrifugation, the supernatant containing V/A-ATPase was applied to a Ni-NTA column. The fractions containing V/A-ATPase were dialyzed against 20 mM Tris-HCl (pH 8.0), 1 mM EDTA for 2 days at 4°C. The combined fraction was applied to a Resource Q column. Eluted fractions were analyzed by SDS-PAGE and the fractions containing V_o_ and V/A-ATPase were concentrated separately using Amicon 100K molecular weight cut-off filters (Millipore).

For nanodisc incorporation, 25 mM DMPC (Avanti) solubilized in 5% DDM was used. The concentrated V_o_ fraction, the scaffold protein MSP1E3D1, and DMPC were mixed in a 1:12:600 molar ratio and incubated for 30 min at room temperature. Then, 200 μL of Bio Beads SM-2 equilibrated with wash buffer (20 mM Tris-HCl, pH8.0, 150 mM NaCl) were added into 500 μL of the protein-lipid mixture. After 2 hr of incubation at 4°C with gentle stirring, an additional 300 μL of Bio Beads was added to the mixture prior to overnight incubation at 4°C to form nanodisc. The supernatant of the mixture containing the nanodisc-V_o_ was applied to a Superdex200 Increase 10/300 column equilibrated in wash buffer. Individual fractions were analyzed by SDS-PAGE and concentrated to ~4 mg/mL. The prepared nanodisc-V_o_ was stored at 4°C and used for cryo-grid preparation within several days.

V/A-ATPase was reconstituted into lipid nanodiscs using the same protocol as that for V_o_, except that 1-Palmitoyl-2-oleoyl-sn-glycero-3-phosphocholine (POPC, Avanti) was used as the lipid during reconstitution. Purified V/A-ATPase solubilized in 0.03% n-Dodecyl-β-D-maltoside (DDM) was mixed with the lipid stock and membrane scaffold protein MSP1E3D1 (Sigma) at a specific molar ratio of V/A-ATPase: MSP: POPC lipid = 1: 4: 520 and incubated on ice for 0.5 hr. 200 μl of Bio-beads SM-2 were added to initiate the reconstitution by removing detergents from the system and the mixture was incubated at 4°C for 3 hr with constant rotation. The bio-beads were removed and the nanodisc mixture applied to a Superdex200 Increase 10/300 column (GE Healthcare) pre-equilibrated in buffer (20 mM Tris-HCl pH8.0, 150 mM NaCl, 2 mM MgCl_2_). Reconstitution was assessed by both size exclusion chromatography and SDS-PAGE. The peak corresponding to the nanodisc-reconstituted V/A-ATPase was immediately used for cryo-EM observation.

### Biochemical analysis

For measurements of proton channel activity, purified Vo was reconstituted into liposomes. L-α-Phosphatidylcholine Type II-S (Sigma-Adrich) was washed repeatedly beforehand to eliminate contamination of K^+^ ions (Soga et al., 2012), and the L-α-Phosphatidylcholine suspension was adjusted to a final concentration of 40 mg/mL in 4 mM Tricin, and 5 mM MgCl_2_ . To 250 μL of L-α-Phosphatidylcholine suspension, 250 μL of a solution containing 8% (w/v) n-octyl-β-D-glucoside (Sigma), and 500 mM KCl were added. Then, 60–80 ng of purified V_o_ was added. After a 30 min incubation at 4°C, 200 μL of Bio Beads SM-2, pre-equilibrated in 2 mM Tricin and 2.5 mM MgCl_2_, were added to the mixture. The bead mixture was gently stirred for 30 min at room temperature. After that, 300 μL of Bio Beads were added to the mixture and incubated for another 2 hr. The supernatant was ultracentrifuged (40 k rpm, 4°C, 30 min) to remove contaminating KCl. The pellet containing reconstituted proteoliposome was re-suspended in 2 mM Tricin and 2.5 mM MgCl_2_. The proteoliposomes were used for proton channel assay immediately. Proton channel activity was detected by the fluorescence quenching of 9-amino-6-chloro-2-methoxyacridine (ACMA) (Thermo Fisher), which changes fluorescence in response to pH reduction inside the proteoliposome. Fluorescence changes were monitored using a spectrofluorometer (FP-6200, JASCO). A 1200 μL aliquot of reaction buffer (2 mM Tricin, pH8.0, 2.5 mM MgCl_2_, 500 mM KCl + NaCl, 1 μL of 30 mg/mL ACMA, 20 μL of proteoliposome) were incubated at 25°C. Proton channel activity was initiated by injection of 1 μL of 0.1 mg/mL valinomycin at the time = 50 s. After 100 s, 1 μL of 0.2 mg/mL carbonyl cyanide-p-trifluoromethoxyphenylhydrazone was added. The initial rate of pH change was estimated from the linear fitting of the initial decay of fluorescence. The membrane potential (ΔΨ) across the liposome membrane was calculated by the Nernst equation, ΔΨ = (kBT/zF)ln([K^+^]_out_/[K^+^]_in_)=59.2 log([K^+^]_out_/[K^+^]_in_) in mV at 25°C, where [K^+^]_out_ was taken to be that of the reaction buffer, and [K^+^]_in_ was 500 mM as in the buffer for proteoliopsome reconstruction.

Protein concentrations of V_o_ were determined from UV absorbances calibrated by quantitative amino acid analysis; 1 mg/ml gave an optical density of 0.56 at 280 nm. Polyacrylamide gel electrophoresis in the presence of SDS or AES was carried out as described previously (Nakano et al., 2008). The proteins were stained with Coomassie Brilliant Blue.

### EM imaging

For cryo-grid preparation, Quanfifoil R1.2/1.3 molybdenum grids were glow discharged by an Ion Bombarder (Vacuum Device) for 1 min. 2.4–2.7 μL of nanodisc-V_o_ were loaded onto the grid and blotted for 9 s with a blot force of 10, wait time of 0 s at 4°C, and 100% humidity using a Vitrobot (FEI). Then, the grid was plunged into liquid ethane without drain time. Cryo-EM movie collection was performed with the CRYOARM200 (JEOL) operating at 200 keV accelerating voltage and equipped with a direct electron detector, K2 Summit (Gatan) in electron counting mode using the data collection software JADAS. The pixel size was 1.1 Å/pix (x5,0000), a total dose of 79.2 e^-^/ Å^2^ (1.32 e^-^/ Å^2^/frame) with a 12 s exposure time (60 frames), and a defocus range of −1.0 to −3.5 μm.

For V/A-ATPase analysis, gold grids were used to reduce beam-induced movement (Russo and Passmore, 2014). A 2.4 μL aliquot of V/A-ATPase sample at 3.5 mg/ml was added to a 1.2 μm hole, 1.3 μm spacing holey gold grid (Quantifoil UltrAuFoil) in a semi-automated vitrification device (Vitrobot, FEI/Thermo Fisher) at 100% humidity, 4°C. The grid was then automatically blotted once from both sides with filter paper for a 9 s blot time. The grid was then plunged into liquid ethane without a delay time. Preparations of the V/A-ATPase were observed with a Titan Krios (FEI/Thermo Fisher) operating at 300 kV acceleration voltage and equipped with a Falcon II (FEI/Thermo Fisher) detector at a magnification of 75,000x with a pixel size of 1.1 Å, set up to capture 34 frames, corresponding to a total dose of 91 e^-^/ Å^−2^ in a defocus range of −2.4 to −3.0 μm.

### Image processing

Image processing was performed using the Relion 3.0.7 software (Zivanov et al., 2018). A total of 5988 cryo-EM movies were collected for isolated V_o_ and 3694 movies collected for V/A-ATPase. All images were subjected to motion correction using the MotionCor2 program (Zheng et al., 2017) followed by contrast transfer function (CTF) estimation using Gctf (Zhang, 2016). Manual selection of the motion-corrected micrographs results in 3268 good isolated V_o_ micrographs and 3084 good V/A-ATPase micrographs. For V_o_, a template for particle auto-picking was generated by 2D classification of particles picked by the LoG (Laplacian of Gaussian) method implemented in the Relion software, while particles were picked manually to generate references for auto-picking for V/A-ATPase. V_o_ and V/A-ATPase particles were picked from each selected micrograph by template-based auto-picking and classified by several rounds of reference-free 2D classification (3.14 × 10^6^ and 0.35 × 10^6^ particles images, respectively). After 2D classifications, 706,617 particles selected for V_o_ and 147,292 particles selected for V/A-ATPase were subjected to several rounds of 3D classification, respectively. The initial model of V_o_ was generated from the V_o_ domain of our previous *T. thermophilus* V/A-ATPase structure (Nakanishi et al., 2018) using UCSF chimera (Pettersen et al., 2004). A total of 175,930 particles selected for V_o_ and 71,196 particles selected for V/A-ATPase assigned into good 3D classes were subjected to 3D auto-refinement followed by CTF refinement of Bayesian polishing. Then, 157,618 V_o_ particles were selected from the polished particles by 2D classification. Another round of 3D auto-refine, CTF refinement, and a final round of masked auto-refine gave a V_o_ map at 3.9 Å resolution and a V/A-ATPase map at 3.6 Å resolution. The resolution was estimated based on the gold standard FSC = 0.143 criterion. However, while the membrane domain was visible it was not well refined in the V/A-ATPase map. This is likely to be due to the structural flexibility between the V_o_ and V_1_ domains in this class. Therefore, focused classification with signal subtraction of the membrane domain was carried out for the V/A-ATPase map to obtain high-quality maps and this gave a near-atomic resolution (3.5 Å resolution) map of the hydrophilic domain (A_3_B_3_DFE_2_G_2_da_sol_).

### Model building

To generate an atomic model for the isolated V_o_ domain, each subunit of the V_o_ complex from the previous structure of the *T. thermophilus* V/A-ATPase (PDBID: 5Y5X) was fitted into the density map as a rigid body. Notably the a-subunit was divided into soluble and transmembrane domains and these domains fitted into the map separately. The rigid body structures were fitted against the density map manually using the COOT software (Emsley et al., 2010). Then, the manually fitted structures were refined using the phenix.real_space_refine program contained in the Phenix suite software (Adams et al., 2010). These processes were performed over several rounds. The geometry of the atomic model built in this study was checked using the MolProbity tool (Table 1; Chen et al., 2010).

**Table 1.** CryoEM data collection, refinement and model statistics.

	TthV/A-ATPase	V_1_EG*da*_sol_	Isolated V_o_
Data collection			
Electron microscope	Titan Krios		CRYOARM200
Electron detector	Falcon II		K2 summit
Magnification	75,000		50,000
Voltage (kV)	300		200
Electron exposure (e^-^/Å^2^)	91		79.2
Defocus range (μm)	2.4–3.0		1.0–3.0
Pixel size (Å)	1.1		1.1
Movie No.	3694		5988
Frame per movie	34		60
Automation software	EPU		JADAS
Data processing			
Total extracted particles	3.5 × 10^5^		3.14 × 10^6^
Total particle after 2D	144,758		706,617
Resolution (Å)	3.6	3.5	3,93
Sharpening B-factor	−81.07	−60.25	−110.87
EMDB ID	30013	30014	30015
Model building and refinement			
Initial models	-	5Y5Y	5Y5X, 1V9M
Building and refinement package	-	COOT, phenix	COOT, phenix
Total atom No.	-	26,631	13,888
Total residue No.	-	3418	1894
Total chain No.	-	8	16
Ligands	-	ADP	-
cc_mask	-	0.85	0.82
Ramachandran favored	-	88.89%	92.75%
Ramachandran outliers	-	0.03%	0.00%
Rotamer outliers	-	11.45%	0.37%
c-beta deviation	-	0	0
CaBLAM outliers	-	5.94%	2.84%
Clashscore	-	6.38	20.01
RMSD bonds (Å)	-	0.006	0.006
RMS angle (°)	-	0.669	0.725
MolProbity score	-	2.73	2.26
PDB ID	-	6LY8	6LY9

Part of the 4.7 Å resolution hydrophilic domain structure of the *T. thermophilus* V/A-ATPase (PDBID: 5Y5Y) was fitted into the map of A_3_B_3_DFE_2_G_2_da_sol_. A rough initial model was refined against the map with the Phenix suite phenix.real_space_refine program (Adams et al., 2010). The initial model was extensively manually corrected residue by residue in the COOT graphics program (Emsley et al., 2010), in particular with respect to side-chain conformations. The peripheral stalks and d-subunit were removed because of a low resolution in these regions. The corrected model was again refined by the phenix.real_space_refine program with secondary structure, and the resulting model manually checked by COOT (Emsley et al., 2010). This iterative process was performed for multiple rounds to correct any remaining errors until the model was in good agreement with the geometry, as reflected by the MolProbity score of 2.21 for isolated V_o_ and 2.75 for A_3_B_3_DFE_2_G_2_da_sol_ .

For model validation against over-fitting, the built models were used for calculation of FSC curves against both half maps, and those were compared with the FSCs of the final models against the final density maps used for model building by the phenix.refine program .

## Data Availability

The density maps and the built models for Tth VoV1, Tth V1 (focused refined), and Tth Vo were deposited in EMDB (EMDB ID; 30013, 30014, and 30015) and PDB (PDB ID; 6LY8 for V1 and 6LY9 for isolated Vo), respectively. All data is available in the main text or the supplementary materials. The following datasets were generated: KishikawaJNakanishiAFurutaAKatoTNambaKTamakoshiMMitsuokaKYokoyamaK2020V/A-ATPase from Thermus thermophilusElectron Microscopy Data BankEMD-30013 KishikawaJNakanishiAFurutaAKatoTNambaKTamakoshiMMitsuokaKYokoyamaK2020V1-ATPase built from cryo-EM map of V/A-ATPase from Thermus thermophilus.Electron Microscopy Data Bank30014 KishikawaJNakanishiAFurutaAKatoTNambaKTamakoshiMMitsuokaKYokoyamaK2020The membrane-embedded Vo domain of V/A-ATPase from Thermus thermophilusElectron Microscopy Data Bank30015 KishikawaJNakanishiAFurutaAKatoTNambaKTamakoshiMMitsuokaKYokoyamaK2020V/A-ATPase from Thermus thermophilus, the soluble domain, including V1, d, two EG stalks, and N-terminal domain of a-subunit.RCSB Protein Data Bank6LY8 KishikawaJNakanishiAFurutaAKatoTNambaKTamakoshiMMitsuokaKYokoyamaK2020The membrane-embedded Vo domain of V/A-ATPase from Thermus thermophilusRCSB Protein Data Bank6LY9

## Additional files

Transparent reporting form
